# The dual role of friendship and antipathy relations in the marginalization of overweight children in their peer networks: The TRAILS Study

**DOI:** 10.1371/journal.pone.0178130

**Published:** 2017-06-07

**Authors:** Kayla de la Haye, Jan Kornelis Dijkstra, Miranda J. Lubbers, Loes van Rijsewijk, Ronald Stolk

**Affiliations:** 1 Department of Preventive Medicine, University of Southern California, Los Angeles, California, United States of America; 2 Interuniversity Center for Social Science Theory and Methodology (ICS) and Department of Sociology, University of Groningen, Groningen, Netherlands; 3 Department of Social and Cultural Anthropology, Autonomous University of Barcelona, Barcelona, Spain; 4 University Medical Center Groningen, University of Groningen, Groningen, Netherlands; Cinvestav-Merida, MEXICO

## Abstract

Weight-based stigma compromises the social networks of overweight children. To date, research on the position of overweight children in their peer network has focused only on friendship relations, and not on negative relationship dimensions. This study examined how overweight was associated with relations of friendship and dislike (antipathies) in the peer group. Exponential random graph models (ERGM) were used to examine friendship and antipathy relations among overweight children and their classmates, using a sub-sample from the TRacking Adolescents’ Individual Lives Survey (*N* = 504, *M* age 11.4). Findings showed that overweight children were less likely to receive friendship nominations, and were more likely to receive dislike nominations. Overweight children were also more likely than their non-overweight peers to nominate classmates that they disliked. Together, the results indicate that positive and negative peer relations are impacted by children’s weight status, and are relevant to addressing the social marginalization of overweight children.

## Introduction

An important challenge for children is developing friendship relations [1, 2], and failing to gain a sense of belonging in the peer group has been associated with poor psychosocial outcomes [3]. Being overweight is one aesthetic feature that can hinder the establishment of friendship relations [4]. Children who experience weight-based stigma have been found to be at an increased risk of social isolation, loneliness, depression, low self-esteem, and reduced quality of life [4]. Moreover, despite current high rates of childhood obesity [5] potentially normalizing excess body weight, obese children seem to be stigmatized even more so than when rates were relatively low [6].

The impact of weight-based stigma on young peoples’ friendships has been well documented. Overweight children are less attractive as friends, receiving fewer friendship nominations than their non-overweight peers [7–11]. As a result of this social exclusion they tend to be found at the periphery of their peer networks [12] despite nominating as many, or more, friends than their non-overweight peers [7, 9, 12]. Moreover, overweight children are likely to select each other as friends [7–10], and may subsequently influence and reinforce each others’ overweight status, because studies have shown that children and their close social ties become more similar in body weight over time [8, 13–15]. Together, these findings highlight the importance of excess body weight as a social marker for the establishment of friendships, and the negative implications of the resulting marginalization of overweight youth by their peers.

Yet, to date, studies of weight-based marginalization have solely focused on *exclusion from friendships*, indicative of a passive form of social exclusion. It is not known if overweight children are also overtly rejected by their peers, whereby peers clearly express dislike of overweight children. Traditionally, rejection has been measured as the extent to which children were nominated as being disliked by their peers [3, 16], however recently it has been conceptualized as a dyadic phenomenon, where it is defined as a negative relationship between pairs of children which can either be unilateral or mutual [17, 18]. Involvement in these relationships of rejection—so-called “antipathies”—has been found to negatively impact the social development of children in ways that are distinct from passive marginalization [19–21]. For example, research on neglected and rejected children have shown overtly marginalized youth are especially at risk for maladjustment [19]. So, overweight might lead to marginalization by having fewer friends, but its impact on youths’ psychosocial development is likely to depend on whether marginalization is also the result of being overtly rejected by peers. A more complete assessment of the marginalization of overweight youth should therefore assess friendship relations as well as examine the extent to which overweight is related to having antipathies in the peer group.

The aim of this study is to examine how overweight is related to both friendship relations and antipathies in children’s peer network. Overweight children are expected to receive fewer friendship nominations, as well as more dislike nominations, relative to their non-overweight peers, with the latter indicative of a more overt process of rejection and marginalization. Consequently, we also expect that overweight children have more friendships with each other. As gender is an important determinant steering friendship preferences [22], this attribute is controlled for. Statistical models for social networks (Exponential Random Graph Models, ERGMs), were used to test for associations between individual (overweight) and relational (friendships, antipathies) variables using a modeling framework that accounts for the complex structure of friendship and antipathy networks and the inherent dependencies within them [23–25].

## Materials and methods

### Sample

A peer nomination subsample was used from the first wave of the TRacking Adolescents’ Individual Lives Survey (TRAILS) cohort, collected between March 2001 and July 2002 [26–28]. The TRAILS sample consisted of preadolescents living in five municipalities (urban and rural) in the north of the Netherlands. The ‘peer subsample’ comprised of school classes with at least ten TRAILS participants, in which peer relations among classmates were assessed. Children in special education or in small schools, and children who repeated or skipped a grade were excluded from this peer subsample [28]. Written consent to participate in the study was obtained from both the parent and child. The study protocols were approved by the Netherland’s national ethics committee ‘Central Committee on Research Involving Human Subjects’ (CCMO).

For the present study, classes with participation rates under 60% were excluded to ensure reliable estimates of friendship and antipathy patterns. This yielded a target sample of 28 school classes in the last year of primary education, with information on friendships and antipathies received of 714 children, including TRAILS participants (*N* = 504) and the non-participating classmates that they nominated (*N* = 210). Information on nominations *received* by non-respondents was retained to gain a more complete representation of the network structure, however the inclusion of non-respondents in our classroom social network data means that their outgoing nominations are coded as missing. This limits our ability to distinguish between unidirectional vs. mutual (reciprocal) friendship and dislike relations, and so our analyses focus on overweight children’s involvement in any (directed or mutual) friendship and dislike relation. Information on all other variables was only available for respondents, resulting in a target sample of 504 children (*M* age: 11.38, *SD* = 0.48, range 10.3 to 12.9; sex: 54.2% girls). There were no significant differences between the analytic sub sample included in this study and other TRAILS participants in terms of body mass index (BMI) or the proportion of participants who were overweight or obese.

### Measures

#### Friendships

Participants nominated an unlimited number of their best friends in their school class. Friends could be any gender and could include classmates not participating in TRAILS. In the Netherlands, children take courses with a fixed group of classmates, therefore classroom-based friendships are likely to be an important segment of their broader friendship networks. Best friend nominations were used to define a directed adjacency matrix representing the friendship network within each school class, where for each directed pair of students *x*_*ij*_ = 1 if student *i* nominated student *j* as a friend.

#### Antipathies

Participants nominated peers they disliked in their class, and similar to friendship relations, the number and gender of nominations was not restricted. Again, dislike nominations were used to define a directed adjacency matrix representing the antipathy network within each school class, where for each directed pair of students *x*_*ij*_ = 1 if student *i* nominated student *j* as someone they dislike.

#### Anthropometry

Height and weight were measured individually by trained research assistants using a SECA 208 stadiometer and a SECA 761 mechanical scale, and used to calculate participants’ BMI (kg/m^2^). Internationally validated age and gender specific BMI cut-off points [29] were used to classify participants as non-overweight or overweight (the latter including overweight and obese classifications).

#### Demographics

Participants reported on their gender (*male / female*) and age.

### Statistical analyses

A two-stage multilevel procedure was applied for the analyses. In the first stage, each network was analyzed separately using ERGMs [23, 25]. The unit of the analysis is the ordered pair of students in a classroom (*x*_*ij*_), and the dependent variable is the observed value of a friendship or dislike tie (1 = present, 0 = absent). As a consequence of the binary nature of the dependent variable, ERGMs are logistic models to predict the probability that a tie exists. As pairs of students are clearly not independent, dependencies in the data are explicitly modeled as structural effects in ERGMs (e.g. reciprocity and transitivity). These structural tendencies were estimated alongside effects testing the hypothesized associations between weight status and friendship or antipathy relations and control-attribute effects, to identify those most likely to explain the structure of the observed networks [24, 30]; that is, the particular configurations of ties that occur more or less than would be expected at chance levels, given the number of nodes and density of the network (and therefore accounting for differences in network size and density across classrooms). ERGMs were fit separately for the friendship and antipathy networks using PNet [31], and a Markov Chain Monte Carlo approach was used to estimate model parameter and standard errors.

To assess relationships between participants’ attributes (e.g., weight status) and their friendship and antipathy relations, three types of effects were included in the models. A “sender effect” represents the association between an attribute and the number of nominations given by participants. A “receiver effect” represents the association between an attribute and the number of nominations received. A “similarity effect” tests whether the probability of a network tie is greater among dyads that have the same score on an attribute. These participant attribute effects were included for weight status (1 = overweight, 0 = non-overweight) and gender (1 = male, 0 = female). To control for potential differences in the network position of classmates who did not participate in the survey, we included a receiver parameter for non-respondents to model their incoming nominations.

The ERGM analyses yield a set of parameter estimates and associated standard errors for each of the classes. In the second stage, these findings were combined in a meta-analysis [32, 33], where class coefficients were split into an average coefficient and a class-dependent deviation. To differentiate between true and error variance, and thus to obtain more precise estimators for the average effects and the variance of the effects across classes, we accounted for the differences in standard errors between classes, such that classes with large standard errors have less influence on the average effect size. Additionally, classes with very large standard errors (> 4) on a parameter were removed from the analysis of the average effect and variance of that parameter, as in these cases the regression coefficient was usually very high as well. The program MLwiN [34] was used for an iterated estimation of the weighted least squares. Average effects across classes are deemed significant at a .05 level when the ratio of the parameter estimate to the standard error exceeds 1.96 [30]. Significant positive parameter estimates indicate the effect is more prevalent than would be expected by chance, given other effects in the model, and the reverse is true for negative estimates. In addition to the average effects across classes, we also report on variance of the effect, which indicates whether classes differ in the extent to which the effect occurs.

## Results

### Descriptive statistics and visualizations

Descriptive statistics for both types of relationships are presented in Table 1. There was an average of 25 participating students per classroom (with a range of 13 to 36). The mean number of friendship nominations given and received was 6.91 (4.45) and 4.81 (2.37), respectively, whereas the mean number of dislike nominations given and received was 3.78 (4.79) and 2.15 (2.24). Approximately 15 percent of the participants were overweight, and there was an average of 2 to 3 overweight children per classroom, with a range of 5.0% to 50.0% of participants that were overweight in each classroom.

**Table 1:** Descriptive statistics.

N total	714
N non-respondents	210
N classes	28
M (SD) number of participants per class	25.5 (6.4)
% male	45.8
M (*SD*) Number of overweight children per class	2.66 (1.63)
M (*SD*) Friendship Nominations given	6.91 (4.45)
M (*SD*) Friendship Nominations received	4.81 (2.37)
M (*SD*) Dislike Nominations given	3.78 (4.79)
M (*SD*) Dislike Nominations received	2.15 (2.24)
M (*SD*) BMI	17.96 (2.99)
% overweight	15.8

*Note*. Data for “Nominations received” were available for both TRAILS respondents and non-respondents. For all other characteristics data were only available for TRAILS respondents.

**Fig 1:**
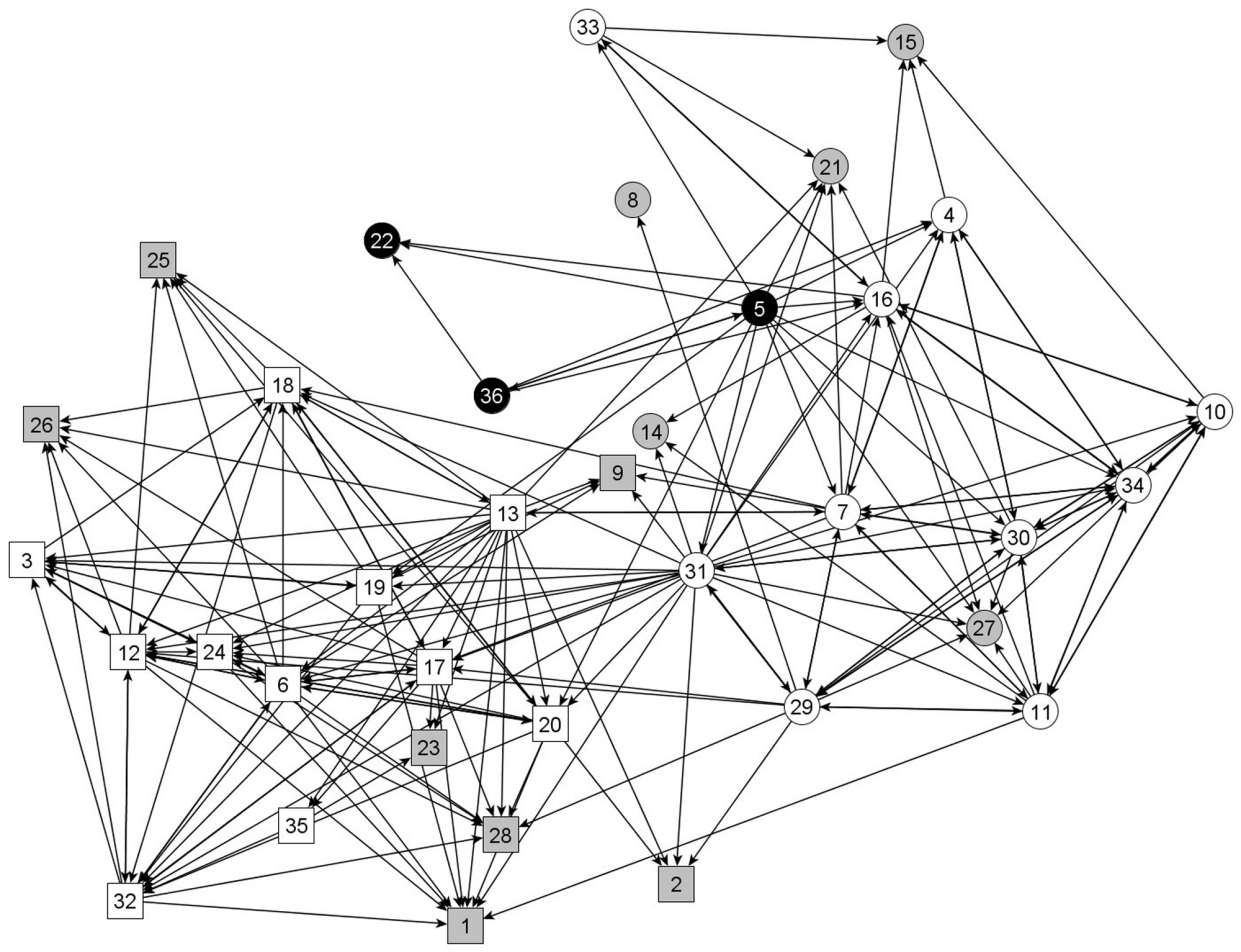
The network of friendship relations in one class. Each node represents one student in the classroom (N = 36), and directed ties represent nominations of friendship. Boys are represented by square nodes, and girls by circles. The black nodes represent the overweight children, the white nodes are non-overweight children, and the grey nodes are non-participants for whom no information on outgoing friendship/dislike nominations or weight status was obtained.

**Fig 2:**
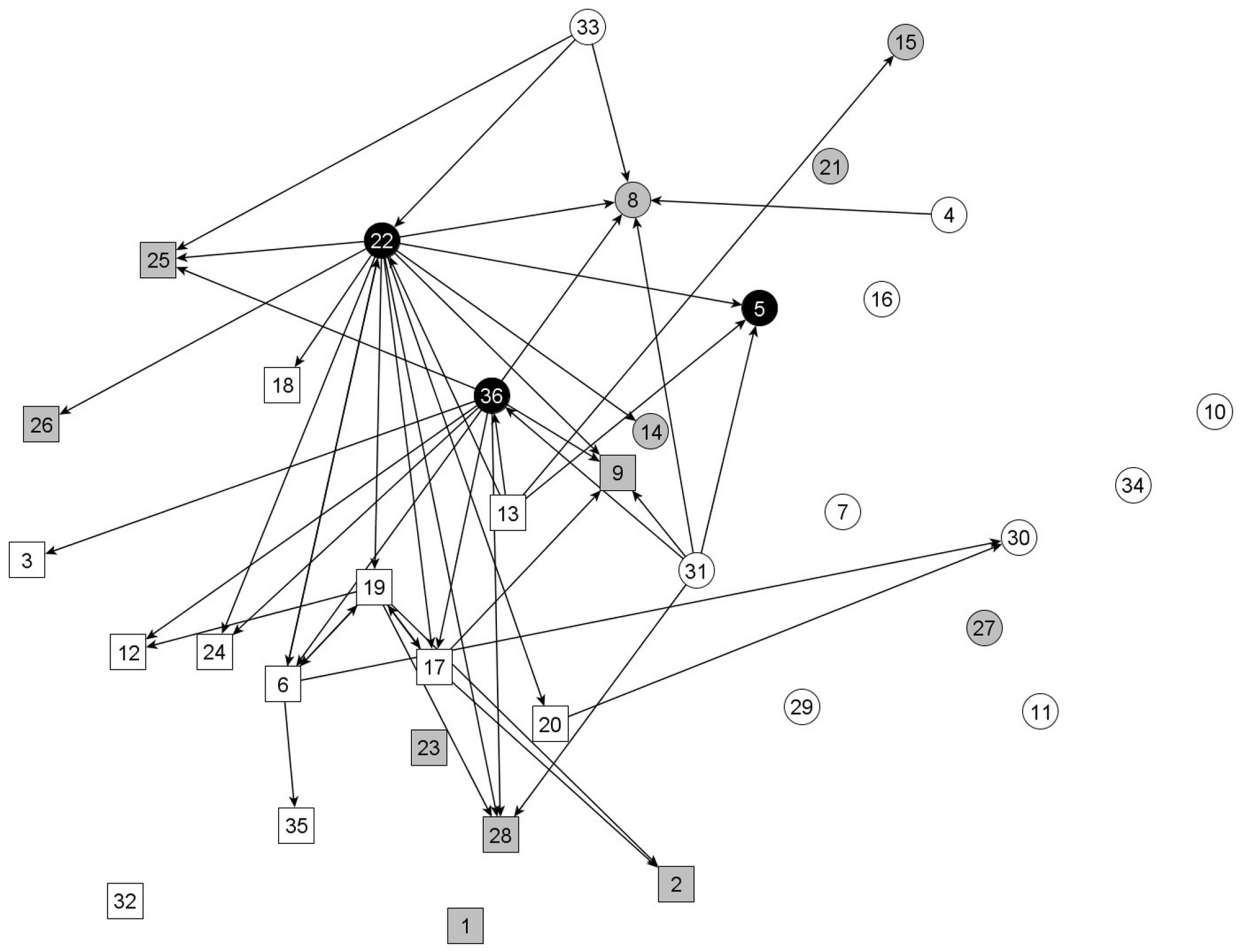
The network of antipathy relations in one class. Each node represents one student in the classroom (N = 36), and directed ties represent nominations of classmates that are disliked. Boys are represented by square nodes, and girls by circles. The black nodes represent the overweight children, the white nodes are non-overweight children, and the grey nodes are non-participants for whom no information on outgoing friendship/dislike nominations or weight status was obtained.

As an example, Fig 1 is a visualization of the friendship network in one classroom, and Fig 2 is a visualization of the antipathy network in the same classroom. Each node represents a student in the classroom and directed lines represent friend or dislike nominations. Nodes are shaped based on gender (squares = boys), and colored based on overweight status and study participation (white = non-overweight participating children, grey = non-participating children, and black = overweight participating children). In this classroom, two of the three overweight girls (nodes 22 and 36) are involved in several antipathy relations, both as senders and receivers, and few friendships with non-overweight children. The third overweight girl (node 5) has nominated more friends than her other overweight classmates, but these are largely unreciprocated, and one of her friendship nominations (to node 22) is reciprocated with a ‘dislike’ nomination.

### Statistical models for friendship networks

Results from the ERGMs fit to the friendship networks (Table 2) show that the observed networks were explained by several processes. The significant structural network effects indicate that there was a strong tendency for friendship ties to be reciprocal (positive reciprocity effect). The negative alternating-in-star and marginally significant positive 2-in-star parameters indicate that participants who received a low number of friendship nominations were most common, and the negative alternating-out-star and positive 2-out-star effects show a tendency for most students to nominate a small number of friends. Additionally, the positive transitive closure and negative multiple connectivity effects showed that “friends of friends” tended to be friends, particularly when there were multiple shared friendships.

**Table 2:** Estimated average effect of model parameters, standard errors, and variance between classes for friendship relations.

Model parameter	*Estimated average*	*SE*	*Estimated variance*
Structural effects			
Reciprocity	1.34**	0.11	0.01
2-in-star	0.04	0.02	0.00
2-out-star	0.15**	0.02	0.00
Alternating-in-star	-0.21	0.19	0.07
Alternating-out-star	-0.90**	0.24	0.34
Transitive closure	0.69**	0.07	0.05
Multiple connectivity	-0.16**	0.02	0.01
Control attribute effects			
Male sender	-1.43**	0.23	0.81
Male received	-1.18**	0.17	0.43
Male similarity	2.95**	0.44	3.14
Non-respondent receiver	0.27**	0.09	0.49
Weight status effects			
Overweight sender	-0.001	0.07	0.03
Overweight receiver	-0.23*	0.10	0.00
Overweight similarity	0.01	0.25	0.00

* *p* < .05.

** *p* < .01.

*Note*. Model parameters were estimated for 23 classes where acceptable ERGM convergence was obtained.

Participants were also more likely to nominate same-gender peers as friends, although this effect varied considerably between classes. Non-respondents also tended to receive more friendship nominations than respondents.

Over and above these effects, overweight children were less likely to be nominated as friends than their non-overweight classmates (negative receiver effect). This effect was negative in 70% of the classes, and significant across classes, indicating its consistency. Weight status was *not* associated with the number of friendship nominations given (non-significant sender effect), and there was no evidence that friends tended to be alike in weight status, as shown by the non-significant overweight similarity effect.

### Statistical models for antipathy networks

Results of the ERGMs fit to the antipathy networks (Table 3) showed a tendency for dislike nominations to be reciprocated, for some participants to receive a higher number of nominations than others (positive 2-instar), and for some participants to make more dislike nominations than others (positive out-star). There was no evidence of triadic closure in antipathy networks, whereby node A disliked node B, node B disliked node C, and node A also disliked node C. This is not surprising, as it indicates that classmates did not have a tendency to cluster in triads or small groups of classmates who all disliked each other.

**Table 3:** Estimated average effect of model parameters, standard errors, and variance between classes for antipathy relations.

Model parameter	*Estimated average*	*SE*	*Estimated variance*
Structural effects			
Reciprocity	0.52**	0.16	0.00
2-in-star	0.64**	0.16	0.30
2-out-star	2.07**	0.12	0.15
Transitive closure	0.02	0.11	0.14
Multiple connectivity	0.01	0.04	0.02
Control attribute effects			
Male sender	0.66**	0.11	0.05
Male received	1.05**	0.13	0.11
Male similarity	-1.19**	0.22	0.37
Non-respondent receiver	0.33**	0.08	0.02
Weight status effects			
Overweight sender	0.18**	0.06	0.00
Overweight receiver	0.53**	0.11	0.00
Overweight similarity	-0.41	0.34	0.00

* *p* < .05.

** *p* < .01.

*Note*. Model parameters were estimated for 21 classes where acceptable ERGM model convergence was obtained, except reciprocity (19 classes), male similarity (19), overweight sender (20 classes), and overweight similarity (11 classes).

The gender effects revealed that relative to girls, boys were more likely to nominate peers that they disliked, and to be nominated as someone who is disliked. The negative gender similarity parameter indicates that antipathy relations were more likely among cross-gender peers. Additionally, non-respondents tended to receive more antipathy nominations than respondents.

Finally, overweight children were more likely to receive dislike nominations than their non-overweight classmates. They were also more likely to nominate peers that they disliked. Specifically, the odds that a dislike tie was present versus absent was 1.65 greater when the receiving student was overweight, and 1.15 greater when the nominator was overweight. The receiver effect was positive in 86% of the classes (18 out of 21 classes), and significantly positive in 4 of these classes. The sender effect was positive in 12 classes, and significantly positive in 4 of these classes. There was no tendency for peers who shared an antipathy relationship to be similar (or dissimilar) in weight status, as shown by the non-significant overweight similarity effect.

## Discussion

Building on previous findings that overweight youth receive fewer friendship nominations [7–10], this study shows that overweight children are not only passively marginalized by their peers by receiving fewer friendship nominations, but they are also overtly rejected by being disliked by more of their peers.

Overweight youth were also found to nominate as many friends as their non-overweight peers; a finding which is in line with longitudinal research showing that marginalization is predominantly driven by overweight youth being excluded by peers who do not reciprocate their extensions of friendship, rather than a result of their own withdrawal [10]. Additionally, this study revealed that overweight children were more likely than their non-overweight peers to dislike their classmates. The combined tendencies for overweight youth to dislike more of their peers, for overweight youth to receive more dislike nominations, and for dislike relationships to be reciprocal, indicates that overweight children are generally more involved in unidirectional and mutual antipathies. This social environment, characterized by fewer friendships and greater antipathies, is likely to put overweight youth at increased risk for psychosocial maladjustment [4]. The resulting social isolation may also promote unhealthy behaviors, such as excessive food intake and decreased participation in sports and physical activities [35], which can lead to further weight gain and thus a cycle of poor physical and social outcomes.

We did not find support for the hypothesis that overweight children would be more likely to befriend other overweight children, which has been shown in other studies [10–12]. Our results may differ because these other studies examined friendship relations among a larger set of peers within grade cohorts or entire schools, while our study examined friendships among a smaller set of peers within classrooms. Hence, opportunities for overweight youth to befriend one another were few with just 2 to 3 overweight students per classroom, on average. It may be that socially marginalized overweight youth seek out friendships with overweight peers who are outside of their class.

### Limitations

Due to the cross-sectional nature of the data, we examined associations between overweight and the structure of these social networks, but could not test for factors driving the selection of network partners or influences of the network on participants; processes which may lead to these observed associations. For example, although we did not find evidence of (dis)similarity in weigh status among peers who shared friendship relations or antipathy relations, the emergence of weight similarity amongst adolescent and adult friends found in other studies [8, 13] could result from longer-term influence processes, whereby weight status assimilates as a result of shared friendship, potentially via similar engagement in obesity-related behaviors [36–38] or shared weight norms [39–41]. Studies that follow youth through these developmental stages are needed to understand the emergence and timing of these processes. It would also be valuable for future longitudinal research to identify social processes and broader structural features of peer networks that give rise to mutual vs. unidirectional friendships and antipathies among overweight youth. For example, mutual antipathies may be established to achieve structural balance within particular triadic network structures, whereby youth dislike the peers that their friends’ dislike; a process that may be socially adaptive and not associated with psychosocial risk for overweight youth [42].

A further limitation of our study is that peer relations were only assessed within classrooms. It is possible that findings may differ with other types of peers (e.g., grade cohort peers, older peers, or neighborhood-based peers).

## Conclusions

Stigmatizing and marginalizing overweight children does not serve to discourage overweight, but rather contributes to their increased risk of negative physical and mental health outcomes [43]. These negative psychological outcomes are not surprising given our findings that overweight children actively seek out friendships, but are marginalized as a result of being passively and overtly rejected by their non-overweight peers. This overt rejection entailed being the target of a greater number of antipathies from their peers, as well as the originator of a greater number of antipathies directed towards their peers. Addressing weight-based stigma, especially amongst non-overweight children, should be a standard component of obesity prevention efforts, with the aim of improving social integration and overall quality of life of overweight children as well as their physical health.

## Abbreviations

BMI: body mass index
ERGM: Exponential random graph models
TRAILS: TRacking Adolescents’ Individual Lives Survey
